# Basal Ganglia-Cortical Circuit Disruption in Subcortical Silent Lacunar Infarcts

**DOI:** 10.3389/fneur.2019.00660

**Published:** 2019-06-25

**Authors:** Haiyan Zhu, Wenxiao Wang, He Li, Kewei Chen, Peng Li, Xin Li, Junying Zhang, Dongfeng Wei, Yaojing Chen

**Affiliations:** ^1^Institute for Cardiovascular Disease, Dongzhimen Hospital Affiliated to Beijing University of Chinese Medicine, Beijing, China; ^2^State Key Laboratory of Cognitive Neuroscience and Learning, Beijing Normal University, Beijing, China; ^3^BABRI Centre, Beijing Normal University, Beijing, China; ^4^Institute of Basic Research in Clinical Medicine, China Academy of Chinese Medical Sciences, Beijing, China; ^5^Computational Image Analysis Lab, Banner Alzheimer's Institute, Phoenix, AZ, United States; ^6^The Laboratory Research Center of Xiyuan Hospital, China Academy of Chinese Medical Sciences, Beijing, China

**Keywords:** silent lacunar infarct, basal ganglia-cortical circuit, cognition, structural connectivity, functional connectivity

## Abstract

To investigate the alterations of basal ganglia (BG)-cortical structural and functional connectivity induced by subcortical silent lacunar infarct (SLI), and their associations with cognitive impairment in SLI subjects. All participants were recruited from communities, including 30 subcortical SLIs and 30 age-, gender-, and education-matched healthy controls. The structural and functional connectivity of BG-cortical circuits using diffusion and resting-state functional magnetic resonance imaging data were obtained. Diffusion abnormalities of the white matter tracts connecting the BG and cortical areas were observed in SLI subjects, including the BG-lateral frontal, BG-orbital frontal, and BG-insula tracts. Multiple regions showed a reduced BG-cortical functional connectivity in SLI patients, including direct connectivities with the BG, such as the BG-limbic, BG-insula, and BG-frontal connectivities, and others that showed no direct causation with the BG, such as the insula-limbic, insula-parietal, and frontal-parietal connectivities. Coupling of structural and functional BG-cortical connectivity was observed in healthy controls but not in SLI patients. Significant correlations between structural and functional BG-cortical connectivity and cognitive performance were demonstrated in SLI patients, indicating the potential use of BG-cortical connectivities as MRI biomarkers to assess cognitive impairment. These findings suggest that subcortical SLIs can impair BG-cortical circuits, and these changes may be the pathological basis of cognitive impairment in SLI patients.

## Highlights

- White matter and functional basal ganglia-cortical circuit disruption in silent lacunar infarcts.
- Focal lesions may spread beyond the sites of initial injury to remote regions throughout specific interconnected networks even at very early and asymptomatic stage.
- Reduced structural and functional BG-cortical connectivity significantly correlated with cognitive performance.

## Introduction

The prevalence of subcortical silent lacunar infarcts (SLIs) has been well-documented. SLIs mainly appear in the basal ganglia (BG) and occur in 11–25% of individuals older than age 65 without psychiatric symptoms or neurological disorders (1, 2). Due to the lack of clinical signs, the danger of SLI is often underestimated. A subcortical SLI is a significant damaging factor to cognition and a key contributor to dementia; in fact, it is considered part of the deleterious dementia pathology despite lacking clinical symptoms (3–5).

Stroke can induce injuries ranging from a single neuron or synapse (at a microscale level) or adjunct neuronal tissues or connections (at a mesoscale level) to different brain regions or even a module composed of multiple brain regions or connections among these modules (at a macroscale level) (6, 7). Through advances in both technology and analysis, brain connectome studies have provided great understanding of the structural and functional disruptions after stroke using multiple measures at various scales in the human brain (7, 8). Brain connectivity disruptions can be observed by the time a stroke is fully diagnosed, however the alterations of connectivity patterns in SLIs in the absence of clinical symptoms remains largely unknown. Recent studies support the assumption that brain functions rely on the connections within network among different brain regions. For SLI patients, an initial subtle impairment to a single brain region may gradually lead to more severe and even irreversible damages to a local or global brain network, along with advancement of the pathological process. Across the whole pathological process, low or partly functioning brain regions with SLI may tend to keep communicating or interacting with the remaining parts of the brain network and gradually spread their harmful functions to adjacent areas (9).

Previous studies have described the anatomy and function of BG-cortical circuits that contribute to human primary and advanced cognitive functions (10, 11). In particular, neuroimaging studies have proven that BG-cortical circuits play an essential role in cognitive function, including executive function and planning and working memory (12, 13). We hypothesized that the disruption in the BG-cortical network is the underlying mechanism of cognitive impairments in SLI patients with BG pathology. This current study, therefore, is designed to determine the cognitive specific BG-cortical structural and functional dysconnectivity in elderly patients with SLI around the BG.

## Methods

### Participants

A total of 60 (30 SLI patients and 30 controls) right-handed, native Chinese participants were used in this study (14). The data were obtained from the Beijing Aging Brain Rejuvenation Initiative (BABRI) database. The Ethics Committee and institutional review board of Beijing Normal University's Imaging Centre for Brain Research approved this study, and all participants gave written informed consent. To be included in this study, participants had to meet the following three additional criteria: (1) a score of at least 24 on the Mini-Mental Status Examination (MMSE); (2) no history of coronary disease, diabetes, nephritis, tumors, gastrointestinal disease or psychiatric illness; and (3) no history of psychoactive medication use. The participants' medical histories and scans were separately reviewed by two experienced neurologists. Clinical judgment of cerebral small vessel disease harmonized with STRIVE criteria (15) when using these standards in clinical practice. We defined lacunar infarcts as round or ovoid lesions of increased signal relative to white matter on T2-weighted or T2-FLAIR images or as decreased attenuation similar to cerebrospinal fluid (CSF)-filled cavities on T1-weighted images that were 3–15 mm in diameter (16). All of the patients had lacunar infarcts around the BG territory, which included the caudate nucleus (*n* = 4), putamen (*n* = 12), globus pallidus (*n* = 2), internal capsule (*n* = 13), and thalamus (*n* = 7). In present study, we recruited elderly subjects from communities, which could decrease the bias of a clinical referral study that tends to recruit patients with more severe impairments. To exclude the confounding effects of white-matter lesions, we therefore only recruited patients with pure lacunar infarcts in the present study.

### Neuropsychological Testing

All participants received a battery of neuropsychological tests assessing general mental status and several cognitive domains. The general mental status was assessed with MMSE [the Mini-Mental-Status Examination-Chinese version, (17)]. The comprehensive neuropsychological battery included the following five cognition domains: (a) Memory function [the Auditory Verbal Learning Test [AVLT] (18), and the Rey-Osterrieth Complex Figure test [ROCF] (recall) (19)]; (b) Visuo-spatial [ROCF-copy (19) and the Clock-Drawing Test [CDT] (20)]; (c) Language [the Category Verbal Fluency Test [CVFT] and the Boston Naming Test [BNT] (21)]; (d) Attention [the Trail Making Test [TMT-A] (22), and the Symbol Digit Modalities Test [SDMT] (23)], and (e) Executive function [the Trail Making Test [TMT-B], (22) and the Stroop Color and Word Test C [SCWT-C] (21)].

The neuropsychological characteristics for each group are presented in Table 1.

**Table 1:** Demographic and neuropsychological measurements.

	**SLI (*n* = 30)**	**HC (*n* = 30)**	***T*-value (X^**2**^)**	***P*-value**
Age	65.96 ± 6.17	63.53 ± 6.10	1.951	0.056
Women (%)	10 (33.3%)	18 (60%)	4.286^a^	0.069
Education	12.10 ± 3.30	10.56 ± 2.73	1.142	0.258
Hypertention (%)	9(30%)	6(20%)	0.800^a^	0.552
Smoking				
non/past/current	24/1/5	27/0/3	1.676^a^	0.241
**GENERAL MENTAL STATUS**				
MMSE	26.43 ± 2.37	27.93 ± 1.55	−2.897	0.005
**Memory function**				
AVLT-delay recall	3.50 ± 2.77	5.10 ± 2.00	−2.559	0.013
AVLT- total	22.03 ± 9.9	29.16 ± 7.18	−3.194	0.002
ROCF-delay recall	12.73 ± 8.53	13.20 ± 5.28	−0.255	0.799
Digit Span	11.77 ± 2.31	11.70 ± 2.12	0.116	0.908
**Visuo-spatial**				
ROCF-Copy	32.50 ± 5.66	33.53 ± 2.62	−0.906	0.369
CDT	24.06 ± 4.14	24.56 ± 3.78	−0.488	0.627
**Language**				
CVFT	40.30 ± 9.48	45.70 ± 11.53	−1.980	0.052
BNT	24.06 ± 3.52	24.13 ± 3.53	−0.073	0.942
**Attention**				
SDMT	30.83 ± 12.79	37.86 ± 9.55	−2.413	0.019
SCWT-B Time(s)	44.66 ± 13.37	38.50 ± 9.48	2.061	0.044
TMT-A time (s)	65.60 ± 26.51	61.80 ± 25.06	0.571	0.571
**Executive function**				
SCWT C-B Time(s)	40.63 ± 23.11	41.83 ± 26.43	−0.187	0.852
TMT-B time(s)	192.83 ± 71.06	188.83 ± 63.54	0.230	0.819

*Values are mean ± SD or Nos. of participants (percentage). The comparisons of neuropsychological scores between the two groups were performed with independent two-sample t-tests*.

a *The p value for gender, hypertension and smoking ratio were obtained using a Chi-square test*.

*MMSE, Mini-Mental State Examination; AVLT, Auditory Verbal Learning Test; ROCF, Rey-Osterrieth Complex Figure test; TMT, Trail Making Test; SDMT, Symbol Digit Modalities Test; SCWT, Stroop Color and Word Test; CDT, Clock-Drawing Test; CVFT, Category Verbal Fluency Test; BNT, Boston Naming Test*.

### Image Acquisition

The MRI data were acquired on a 3.0T Siemens Trio Tim MRI scanner at the Imaging Center for Brain Research, Beijing Normal University. Each participant laid in the supine position with the head snugly fixed by a belt and foam pads to minimize head motion. The following procedures were employed to acquire each set of MRI images. (1) T2-weighted images (TR = 5,000 ms, TE = 105 ms, slice thickness = 3 mm, flip angle = 150°, number of slices = 33) and T2-FLAIR images (TR = 9,000 ms, TE = 81 ms, slice thickness = 3 mm, flip angle = 150°, number of slices = 25) were acquired. (2) T1-weighted, sagittal 3D magnetization-prepared rapid gradient echo (MP-RAGE) sequences were acquired and covered the entire brain [176 sagittal slices, repetition time (TR) = 1,900 ms, echo time (TE) = 3.44 ms, slice thickness = 1 mm, flip angle = 9°, inversion time = 900 ms, field of view (FOV) = 256 × 256 mm^2^, acquisition matrix = 256 × 256]. (3) Two sets of diffusion tensor imaging (DTI) data scans were acquired for every subject and then averaged during the data processing. DTI images covering the whole brain were acquired using a single-shot, twice-refocused, diffusion-weighted echo planar imaging sequence [TR = 9,500 ms; TE = 92 ms; 30 diffusion-weighted directions with a b-value of 1,000 s/mm^2^, and a single image with a b-value of 0 s/mm^2^; slice thickness = 2 mm; no inter-slice gap; 70 axial slices; matrix size = 128 × 128; FOV = 256 × 256 mm^2^; voxel size = 2 × 2 × 2 mm^3^]. (4) During the single-run resting acquisition, subjects were instructed to stay awake, relax with their eyes closed and remain as motionless as possible. Resting functional images were collected using an echo-planar imaging sequence (TE = 30 ms, TR = 2,000 ms, flip angle = 90°, 33 axial slices, slice thickness = 3.5 mm, acquisition matrix = 64 × 64, FOV = 200 × 200 mm^2^). The resting acquisition lasted for 8 min, and 240 image volumes were obtained.

### Data Preprocessing

The DTI data preprocessing and fiber tracking were performed using DTI Studio (http://cmrm.med.jhmi.edu). The preprocessing comprised the following steps: eddy current and motion artifact correction of DTI data, estimation of diffusion tensor, fractional anisotropy (FA) calculation, and diffusion tensor tractography.

Functional data were preprocessed and statistically analyzed using SPM8 package (http://www.fil.ion.ucl.ac.uk/spm/software/). Preprocessing procedures included slice timing, within-subject interscan realignment to correct possible movement, spatial normalization to a standard brain template in the Montreal Neurological Institute coordinate space, resampling to 3 × 3 × 3 mm^3^, and smoothing with an 8 mm full-width half-maximum Gaussian kernel. Finally, the functional data were high-pass filtered with a cutoff frequency of 0.01 Hz, whereas resting functional imaging data were processed with linear detrending and 0.01–0.08 Hz bandpass filtering.

### Structural Connectivity Analyses

Fiber tracking was performed using the Fiber Assignment by Continuous Tracking algorithm (24). The tracking procedure was stopped with an FA threshold of 0.2 and a track turning angle threshold of 45°. The fibers of interest were selected by designating manually defined ROIs and using the three logical operators AND, OR, and NOT. Our analysis of white matter structure was focused on tracts connecting the BG and cortical regions. As a result, the individual cerebrum in the native space were remerged into bilateral BG and 22 cortical and subcortical regions according to an automated anatomical labeling atlas (11 regions for each hemisphere; Table S1). For each hemisphere, we studied the structural connections between the BG and each of 11 ipsilateral regions. The average FA value for each fiber bundle of interest was calculated.

### Functional Connectivity Analysis

Most previous studies showed that patients with SLI in the BG, although clinically normal, already exhibited cognitive decline. The cognitive impairment resulting from SLI is a complex process; we mainly focused on the changes in the organization of the cognitive specific BG-cortical network from a previous meta-analysis (25). The regions of interest (ROI) included 20 regions as detailed in Table S2, such as the caudate head (Cau.head), the thalamus (Tha), the red nucleus (RN), the insula (INS), the cingulate gyrus (CG), the precentral gyrus (PreCG), the inferior frontal gyrus (IFG), the superior parietal lobule (SPL), the inferior parietal lobule (IPL), and Brodmann areas 6, 9, and 46, which are portions of the middle frontal gyrus (MFG.BA6, MFG.BA9, and MFG.BA46). For each subject, the time series in each ROI was obtained by averaging the functional MRI time series across all voxels over a given ROI. To regress out the nuisance covariates, time series were corrected for patient movement, the global mean signal, the white matter signal and the cerebrospinal fluid signal. The Pearson's correlation coefficient of the ROI signal time course in the brain was computed. Specifically, we defined the functional connectivity between two regions as the weight of the network edges. Thus, for each participant, we constructed the weighted BG-cortical network that was represented by a symmetric 20 × 20 matrix.

### Statistical Analysis

#### Demographic and Neuropsychological Measurements

Between-group comparisons were performed using a χ^2^ or two-sample independent *t*-test, depending on the variable type and distribution.

#### Structural Connectivity

For the BG-cortical tract alterations, we performed a two-sample *t*-test to compare the FA value between groups for each tracked fiber.

#### Functional Connectivity

Prior to group comparisons, the correlation coefficients were converted to z-scores using the Fisher r-to-z transformation. To test the between-group differences, two-sample *t*-tests were performed on each transformed *z*-value. For the multiple nodal efficiencies, we applied the false discovery rate (FDR) procedure to correct the multiple comparisons at a *q*-value of 0.05.

Finally, we investigated the relationship between structural and functional BG-cortical connectivity and between BG-cortical connectivity and cognitive performance for SLI and control groups, respectively. Of note, the correlation analyses were performed only among the structural connectivity, functional connectivity, and cognitive scores.

## Results

### Demographic and Clinical Data

There were no significant differences in age, years of education, smoking habits, or medical history of hypertension between SLI and healthy groups. In the SLI patients, cognitive function in the domains of general mental status, memory, and attention were significantly worse than controls (Table 1).

### Structural BG-Cortical Connectivity

**Figure 1:**
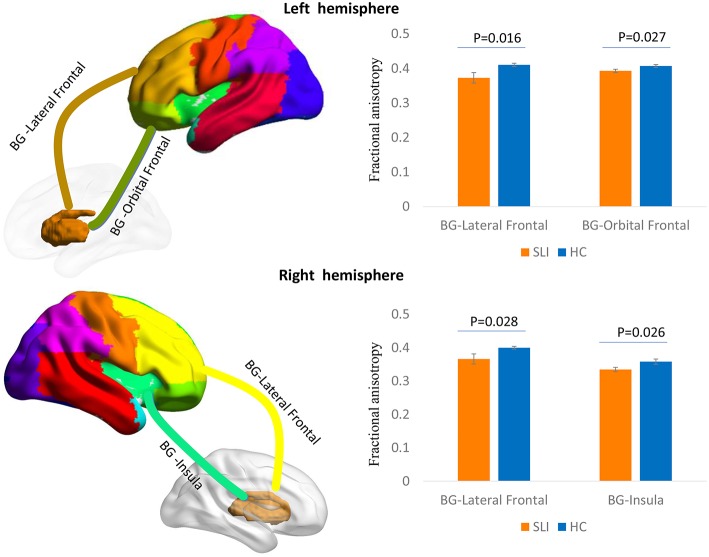
Comparison of structural BG-cortical connectivity between SLI patients and controls. White matter tracts connecting the BG to the lateral frontal area and orbital frontal cortex in the left hemisphere and to the lateral frontal area and insula in the right hemisphere are represented in a 3D brain model. The bar charts show the average FA ± SEM within each tract compared between SLI patients (orange) and controls (blue). BG, basal ganglia; SLI, silent lacunar infarct; FA, fractional anisotropy; SEM, standard error of the mean.

Multiple white matter tracts connecting nodes between BG and cortical regions showed significantly lower FA values in SLI patients, especially for the connections between the BG and the bilateral lateral frontal area, left orbital frontal cortex, and right insula (Figure 1).

### Functional BG-Cortical Network Connectivity

**Figure 2:**
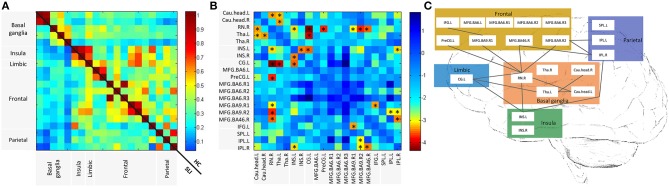
Altered BG-cortical functional connectivities in SLI patients. **(A)** Mean functional connectivity in SLI and HC groups. The Color bar indicates the correlation values for each region pair. **(B)** Differences between SLI and HC in BG-cortical connectivity. Color bar indicates altered functional connectivity values (SLI minus HC) for each region pair. Blue cells represent significant correlation differences between ROIs (*t*-test, FDR-corrected, *q* < 0.05). **(C)** Brain map representation with altered correlations in the BG-cortical network. Significantly decreased connections in SLI patients are shown. There were no increased connections observed in SLI patients. BG, basal ganglia; SLI, silent lacunar infarct; FA, fractional anisotropy; HC, healthy control, FDR, false discovery rate; ROI, regions of interest.

We investigated cognitive specific BG-cortical network connectivity between SLI patients and healthy controls. As shown in Figure 2A, BG-cortical intrinsic FC was decreased in SLI patients compared to controls. The majority of BG-cortical network FCs were significantly lower in SLI patients with BG lesions, including decreases within BG areas (Cau.head.L-RN.R, Cau.head.L-Tha.L, Cau.head.R-Tha.L), between the BG and limbic regions (Tha.L-CG.L, Tha.R-CG.L), between the BG and insula (RN.R-INS.L), and between the BG and frontal regions (RN.R-PreCG.L, RN.R-MFG.BA9.R1, RN.R-MFG.BA9.R02, RN.R-MFG.BA46.R), and connectivity showed no direct causation with the BG, including the insula and limbic region (INS.L-CG.L), between the insula and parietal region (INS.L-IPL.R), between the frontal and parietal regions (MFG.BA9.R2-IPL.L, MFG.BA9.R2-IPL.R), within the insula (INS.L-INS.R), and within the frontal regions (MFG.BA9.R1-IFG.L; Figures 2B,C, FDR-corrected, *q* < 0.05).

### Relationship Between Structural and Functional BG-Cortical Connectivity

We found significant positive correlations between BG-cortical white matter integrity and functional connectivity in controls, but not in the SLI patients (Table 2). The significant correlation results can be corrected for multiple comparisons and therefore should be regarded as exploratory in nature.

**Table 2:** Structural and functional BG-cortical connectivity correlations.

			**Functional connectivity**					
			**Cau.head.L-Tha.L**	**Cau.head.R-Tha.L**	**RN.R-MFG.BA9.R1**	**INS.L-INS.R**	**MFG.BA9.R1-IFG.L**	**MFG.BA9.R2-IPL.R**
White matter integrity	BG-Lateral Frontal.L	SLI	*r =* −0.17 *p =* 0.396	*r =* 0.143 *p =* 0.485	*r =* 0.069 *p =* 0.736	*r =* −0.34 *p =* 0.094	*r =* −0.095 *p =* 0.646	*r =* 0.178 *p =* 0.385
		HC	*r =* 0.058 *p =* 0.763	*r =* 0.086 *p =* 0.653	*r =* 0.098 *p =* 0.607	***r=*** **0.440** ***p=*** **0.014**	***r=*** **0.390** ***p=*** **0.033**	***r=*** **0.406** ***p=*** **0.026**
	BG-Lateral Frontal.R	SLI	*r =* −0.12 *p =* 0.573	*r =* 0.067 *p =* 0.744	*r =* −0.034 *p =* 0.869	*r =* −0.106 *p =* 0.607	*r =* 0.011 *p =* 0.958	*r =* 0.127 *p =* 0.535
		HC	*r =* −0.197 *p =* 0.297	*r =* −0.070 *p =* 0.714	*r =* −0.081 *r =* 0.670	***r=*** **0.386** ***p=*** **0.034**	*r =* 0.165 *p =* 0.382	*r =* 0.021 *p =* 0.911
	BG-Insul.R	SLI	*r =* −0.042 *p =* 0.835	*r =* −0.182 *p =* 0.363	*r =* −0.227 *p =* 0.255	*r =* 0.189 *p =* 0.343	*r =* −0.172 *p =* 0.391	*r =* −0.212 *p =* 0.289
		HC	***r=*** **0.374** ***p=*** **0.04**	***r=*** **0.441** ***p=*** **0.014**	***r=*** **0.383** ***p=*** **0.037**	*r =* 0.0143 *p =* 0.450	*r =* −0.225 *p =* 0.232	*r =* 0.093 *p =* 0.626

*Cau.head, Caudate head; Tha, Thalamus (medial dorsal nucleus); RN, Red nucleus; TMT, Trail Making Test; MFG, Middle frontal gyrus; INS, Insula; IFG, Inferior frontal gyrus; IPL, Inferior parietal lobule; L, Left hemisphere; R, Right hemisphere; BA, Brodmann area. Bold values indicate Functional connectivity*.

### Correlation With Cognitive Function

The structural and functional BG-cortical connectivity measures were correlated with the performance on neuropsychologic tests. For structural connectivity, correlation analyses indicated that the FA value of the BG-Insul.R was positively correlated with the symbol digit modalities test (SDMT) performance and that the FA in the BG-Lateral Frontal.L was significant associated with performance on the auditory verbal learning test (AVLT)-delay recall, the AVLT-total, and the Stroop color and word test part B (SCWT-B) in the SLI patients but not in the controls (Table 3). In addition, higher functional BG-cortical connectivity was significantly correlated with better test performance on the MMSE, the AVLT-delay recall, the AVLT-total, the SCWT-B, and the SDMT in both SLI patients and controls.

**Table 3:** Correlation between BG-cortical connectivity and cognitive function.

			**Cognitive function**				
			**MMSE**	**AVLT-delay recall**	**AVLT-total**	**SDMT**	**SCWT-B Time**
White matter integrity	BG-Lateral Frontal.L	SLI	*r =* −0.27 *p =* 0.18	***r=*** **−0.47** ***p=*** **0.01**	***r=*** **−0.04** ***p=*** **0.03**	*r =* −0.33 *p =* 0.10	***r=*** **0.45** ***p=*** **0.02**
		HC	*r =* 0.08 *p =* 0.67	*r =* 0.32 *p =* 0.08	*r =* 0.12 *p =* 0.53	*r =* 0.09 *p =* 0.64	*r =* −0.21 *p =* 0.27
	BG-Insul.R	SLI	*r =* 0.16 *p =* 0.44	*r =* 0.19 *p =* 0.35	*r =* 0.19 *p =* 0.33	***r=*** **0.40** ***p=*** **0.04**	*r =* −0.15 *p =* 0.45
		HC	*r =* −0.28 *p =* 0.14	*r =* 0.21 *p =* 0.27	*r =* 0.12 *p =* 0.52	*r =* 0.33 *p =* 0.08	*r =* −0.18 *p =* 0.33
Functional connectivity	Cau.head.L-RN.R	SLI	*r =* 0.22 *p =* 0.25	*r =* 0.31 *p =* 0.10	*r =* 0.29 *p =* 0.12	*r =* 0.06 *p =* 0.76	*r =* −0.09 *p =* 0.63
		HC	***r=*** **0.38** ***p=*** **0.03**	*r =* 0.35 *p =* 0.06	*r =* 0.26 *p =* 0.17	*r =* 0.05 *p =* 0.78	*r =* −0.17 *p =* 0.36
	Cau.head.L-Tha.L	SLI	*r =* 0.23 *p =* 0.22	*r =* 0.23 *p =* 0.23	*r =* 0.27 *p =* 0.15	*r =* 0.08 *p =* 0.69	*r =* −0.27 *p =* 0.16
		HC	***r=*** **0.62** ***p*** **<** **0.0001**	*r =* 0.14 *p =* 0.45	*r =* 0.17 *p =* 0.36	*r =* 0.01 *p =* 0.94	*r =* −0.10 *p =* 0.58
	Cau.head.R-Tha.L	SLI	*r =* 0.08 *p =* 0.68	*r =* −0.05 *p =* 0.81	*r =* −0.04 *p =* 0.82	*r =* −0.23 *p =* 0.23	*r =* 0.10 *p =* 0.61
		HC	***r=*** **0.52** ***p=*** **0.002**	*r =* 0.03 *p =* 0.87	*r =* −0.02 *p =* 0.93	*r =* −0.04 *p =* 0.85	*r =* −0.13 *p =* 0.50
	RN.R-CG.L	SLI	*r =* 0.35 *p =* 0.06	*r =* −0.05 *p =* 0.78	*r =* 0.03 *p =* 0.87	*r =* −0.05 *p =* 0.78	*r =* −0.01 *p =* 0.97
		HC	***r=*** **0.51** ***p=*** **0.003**	*r =* 0.26 *p =* 0.17	*r =* 0.12 *p =* 0.51	*r =* −0.06 *p =* 0.74	*r =* −0.002 *p =* 1.0
	RN.R-PreCG.L	SLI	*r =* 0.17 *p =* 0.38	*r =* −0.02 *p =* 0.91	*r =* −0.05 *p =* 0.79	*r =* 0.11 *p =* 0.57	*r =* −0.08 *p =* 0.68
		HC	***r=*** **0.38** ***p=*** **0.04**	*r =* 0.02 *p =* 0.92	*r =* 0.03 *p =* 0.89	*r =* 0.02 *p =* 0.92	*r =* −0.29 *p =* 0.13
	RN.R-MFG.BA46.R	SLI	***r=*** **0.43** ***p=*** **0.02**	*r =* 0.18 *p =* 0.35	*r =* 0.07 *p =* 0.69	*r =* 0.12 *p =* 0.51	*r =* 0.04 *p =* 0.85
		HC	*r =* 0.26 *p =* 0.16	*r =* 0.12 *p =* 0.53	*r =* −0.08 *p =* 0.69	*r =* 0.06 *p =* 0.75	*r =* −0.33 *p =* 0.08
	Tha.L-CG.L	SLI	*r =* 0.12 *p =* 0.53	*r =* 0.10 *p =* 0.59	*r =* 0.15 *p =* 0.43	*r =* 0.02 *p =* 0.91	*r =* −0.25 *p =* 0.18
		HC	***r=*** **0.42** ***p=*** **0.02**	*r =* 0.03 *p =* 0.89	*r =* 0.19 *p =* 0.33	*r =* 0.13 *p =* 0.49	*r =* −0.18 *p =* 0.33
	INS.L- INS.R	SLI	*r =* 0.35 *p =* 0.06	*r =* 0.31 *p =* 0.10	*r =* 0.30 *p =* 0.11	*r =* 0.31 *p =* 0.09	***r=*** **−0.37** ***p=*** **0.04**
		HC	*r =* 0.1 *p =* 0.33	***r=*** **0.38** ***p=*** **0.04**	*r =* 0.25 *p =* 0.19	***r=*** **0.36** ***p=*** **0.05**	***r=*** **−0.401** ***p=*** **0.03**
	INS.L-CG.L	SLI	*r =* 0.29 *p =* 0.12	***r=*** **0.53** ***p=*** **0.002**	***r=*** **0.58** ***p=*** **0.0008**	*r =* 0.27 *p =* 0.15	***r=*** **−0.41** ***p=*** **0.02**
		HC	*r =* 0.13 *p =* 0.48	*r =* 0.03 *p =* 0.88	*r =* 0.12 *p =* 0.53	*r =* 0.08 *p =* 0.66	*r =* −0.16 *p =* 0.41
	INS.L-IPL.R	SLI	*r =* 0.32 *p =* 0.09	*r =* 0.33 *p =* 0.08	*r =* 0.35 *p =* 0.06	***r=*** **0.41** ***p=*** **0.02**	*r =* −0.30 *p =* 0.11
		HC	*r =* 0.24 *p =* 0.20	*r =* −0.07 *p =* 0.72	*r =* 0.01 *p =* 0.94	*r =* 0.03 *p =* 0.89	*r =* −0.19 *p =* 0.31
	MFG.BA9.R2-IPL.L	SLI	*r =* −0.06 *p =* 0.75	*r =* 0.02 *p =* 0.92	*r =* 0.04 *p =* 0.84	*r =* 0.10 *p =* 0.60	*r =* −0.25 *p =* 0.17
		HC	*r =* −0.003 *p =* 0.98	*r =* 0.02 *p =* 0.91	*r =* 0.10 *p =* 0.61	*r =* 0.34 *p =* 0.07	***r=*** **−0.43** ***p=*** **0.02**

*MMSE, Mini-Mental State Examination; AVLT, Auditory Verbal Learning Test; SDMT, Symbol Digit Modalities Test; SCWT, Stroop Color and Word Test; Cau.head, Caudate head; Tha, Thalamus (medial dorsal nucleus); RN, Red nucleus; TMT, Trail Making Test; MFG, Middle frontal gyrus; INS, Insula; IFG, Inferior frontal gyrus; IPL, Inferior parietal lobule; L, Left hemisphere; R, Right hemisphere; BA, Brodmann area. Bold values indicate Functional connectivity*.

## Discussion

The focus of this study was to evaluate small lesion-induced changes in structural and functional connectivity and network topology in the BG-cortical system in patients with subcortical SLI. SLI disrupted the structural integrity of the BG-cortical tracts, including the BG-frontal areas and the BG-insula integrity. We found that SLI leads to damage of direct functional connectivity with BG regions, such as between the BG and frontal, limbic regions and insula connectivity, and remote connectivity, such as to the frontal and parietal areas. Moreover, structural and functional BG-cortical connectivity were correlated in controls but not in SLI patients. We also found a significant association between structural and functional BG-cortical connectivity and several cognitive scores, including performance on the MMSE, the AVLT-delay recall, the AVLT-total, the SCWT-B, and SDMT. Therefore, studying the BG in SLI patients provides an opportunity to investigate the impact of information exchange in the BG-cortical network on cognitive decline.

BG, as an important structural region of the brain, is mainly involved in motor control and cognitive function (26, 27). However, due to the complexity of the molecular and anatomical structure of the BG, neurons in the BG region are more vulnerable to ischemia and toxicity injury (28). In particular, our previous study showed gray matter volume loss in insula, anterior cingulate cortex, caudate and superior temporal pole and connectivity reduced in SLI patients (14). All these evidence provide a solid theoretical basis for this study on SLI patients located in the BG region.

Patients with lacunar infarcts have more severe white matter changes than patients with non-lacunar infarcts among patients with ischemic stroke (29), which is associated with executive dysfunction. Our previous studies have reported that SLIs in the BG region led to local and remote white matter integrity damages (30) and topological alterations of the whole brain white matter network (31), suggesting that BG-frontal and BG-insula integrity are influenced by SLIs. Augustine and colleagues have shown that the BG in primates and humans has efferent and afferent fibers with the insula and prefrontal cortex (32). Additionally, the prefrontal areas consist of a number of modules, which seem to provide multiple subloops of the BG-thalamocortical connections in non-human primates (33, 34). Several investigators have demonstrated that BG lesions impair several cognitive deficits associated with prefrontal activity (35, 36).

We also assessed the intrinsic connectivity within the cognitive specific BG-cortical network. In SLI, a majority of connectivities including the BG-limbic, BG-insula, BG-frontal, insula-limbic, insula-parietal, frontal-parietal connectivities, and the connectivity between BG areas showed disconnection. By applying a longitudinal and lesion-restricted approach, a recent study observed secondary neurodegeneration in remote cortices after a subcortical stroke (37). Considering the network perspective, the BG could modulate advanced cognitive function through cognitive-related functional networks (14). Furthermore, surgery that targets the BG-thalamic cortical loop can restore normal cortical activity associated with behavioral and mood disorders (38, 39). Beneficial attempts to treat psychiatric disease by modulating BG-cortical loops suggested that neurological and psychiatric diseases share essential neurobiological mechanisms. This theory can explain why damage to the BG and related nuclei always affects those brain regions that belonging to the same non-motor BG-cortical loops. Our findings also supported the notion that subcortical SLI not only affected adjacent regions but also the regions in BG-cortical circuits.

Significant correlations between structural and functional BG-cortical connectivity in controls, supporting the notion “functional connectivity reflects anatomical connectivity.” The decoupling of structural and functional connectivity were observed in SLI patients may due to several pathological changes. Decreased perfusion in penetrating arteries may lead to an SLI (40). Feng et al. have shown that SLIs tend to occur in the radiation crown and the BG (41). In the early stages of lacunar infarction, acute ischemic changes can occur in neurons. Then, an influx of macrophages occurs in the subacute stages, followed by neuronal death and glial cell proliferation. In addition, SLI also may have distant effects, such as Wallerian, retrograde or post-synaptic degeneration (42). The pathological damage caused by the combination of neuronal death, gliosis and degeneration are not synchronized and not limited to the lesion itself. Structural and functional connectivity was decoupled in SLI patients.

A recent study in a stroke cohort showed that advanced cognitive functions such as visual and verbal memory deficits are better predicted by functional connectivity than by the lesion location, and primary functions such as visual and motor deficits are better predicted by the lesion location than by functional connectivity (43). This finding may support the notion that stroke is a disease of brain connectivity, which is being supported by an increasing number of studies (44–46). There was a significant correlation between BG-cortical connectivity and general mental status, memory, and attention performance. Robust evidence has shown that the existence of BG-cortical loops achieves connectivity of the BG to the cerebral cortex and can be subdivided into motor, associative, and limbic domains (47). Therefore, the dysfunction of these BG-based circuits can lead to movement disorders, cognitive impairment, and emotional disorders. It is worth noting that the BG and its dopaminergic projections participate in some cognitive activities. A BG injury could potentially induce changes in these circuits and gradually result in cognitive impairment.

Despite the interesting findings in the present study, a few limitations need to be addressed. First, a longitudinal dataset is highly desired to verify the current results. Next, the heterogeneity of lesion location may result in different cognitive impairment and functional disconnection patterns. Therefore, stratification of patients according to lesion location should be considered for future work.

In summary, this study demonstrated a structural and functional disconnectivity of the BG-cortical network in the elderly with silent small lesions around the BG. Focal lesions can spread beyond the sites of initial injury to remote regions throughout specific interconnected networks. Both structural and functional BG-cortical connectivity strength were predictive of cognitive dysfunction. Taken together, the study opens up a new avenue for investigating lesion-induced network dysfunction, highlighting the importance of early damage to the structural and functional connectivity as a potential manifestation of preclinical small vessel disease.

## Data Availability

All datasets generated for this study are included in the manuscript and/or the Supplementary Files.

## Ethics Statement

This study was carried out in accordance with the recommendations of the institutional review board of BNU imaging center for brain research, national key laboratory of cognitive neuroscience and learning, BNU with written informed consent from all subjects. All subjects gave written informed consent in accordance with the Declaration of Helsinki. The protocol was approved by the institutional review board of BNU imaging center for brain research, national key laboratory of cognitive neuroscience and learning, BNU.

## Author Contributions

HZ, WW, and HL designed the entire study and contributed to the study equally. HZ also analyzed the cognitive data. WW and HL analyzed the neuroimage data and wrote the main part of the manuscript. KC guided the data analysis and participated in the revision of the article. PL and XL participated in data analysis and discussion. JZ and DW wrote part of the article. YC participated in the coordination of the study and reviewed the manuscript. All authors read and approved the final manuscript.

### Conflict of Interest Statement

The authors declare that the research was conducted in the absence of any commercial or financial relationships that could be construed as a potential conflict of interest.
