# Supplementary material for: Basal Ganglia-Cortical Circuit Disruption in Subcortical Silent Lacunar Infarcts
Source: Front Neurol. 2019 Jun 25;10:660. doi: 10.3389/fneur.2019.00660 (PMC6603169; doi:10.3389/fneur.2019.00660)
Supplement: Supplementary file 1 [file Table_1.DOCX]

**Supplementary material**

**Table S1** Remerged ROIs based on AAL template for structural connectivity analysis.

| **AAL index** | **Abbr.** | **Regions** | **New index** | **Remerged ROI** |
| --- | --- | --- | --- | --- |
| 1,2 | PreCG | Precental gyrus | 1,2 | Central region |
| 3,4 | SFGdor | Superior frontal gyrus, dorsolateral | 3,4 | Frontal lobe_lateral |
| 5,6 | ORBsup | Superior frontal gyrus, orbital part | 5,6 | Frontal lobe_orbital |
| 7,8 | MFG | Middle frontal gyrus | 3,4 | Frontal lobe_lateral |
| 9,10 | ORBmid | Middle frontal gyrus, orbital part | 5,6 | Frontal lobe_orbital |
| 11,12 | IFGoperc | Inferior frontal gyrus, opercular part | 3,4 | Frontal lobe_lateral |
| 13,14 | IFGtriang | Inferior frontal gyrus, triangular part | 3,4 | Frontal lobe_lateral |
| 15,16 | ORBinf | Inferior frontal gyrus, orbital part | 5,6 | Frontal lobe_orbital |
| 17,18 | ROL | Rolandic operculum | 1,2 | Central region |
| 19,20 | SMA | Supplementary motor area | 7,8 | Frontal lobe_medial |
| 21,22 | OLF | Olfactory cortex | 5,6 | Frontal lobe_orbital |
| 23,24 | SFGmed | Superior frontal gyrus, medial | 9,10 | Frontal lobe_medial |
| 25,26 | ORBsupmed | Superior frontal gyrus, medial orbital | 5,6 | Frontal lobe_orbital |
| 27,28 | REC | Gyrus rectus | 5,6 | Frontal lobe_orbital |
| 29,30 | INS | Insula | 11,12 | Insula |
| 31,32 | ACG | Anterior cingulate and paracingulate gyri | 13,14 | Limbic lobe |
| 33,34 | DCG | Median cingulate and paracingulate gyri | 13,14 | Limbic lobe |
| 35,36 | PCG | Posterior cingulate gyrus | 13,14 | Limbic lobe |
| 37,38 | HIP | Hippocampus | 13,14 | Limbic lobe |
| 39,40 | PHG | Parahippocampal gyrus | 13,14 | Limbic lobe |
| 41,42 | AMYG | Amygdala | 13,14 | Limbic lobe |
| 43,44 | CAL | Calcarine fissure and surrounding cortex | 15,16 | Occipital lobe_medial |
| 45,46 | CUN | Cuneus | 15,16 | Occipital lobe_medial |
| 47,48 | LING | Lingual gyrus | 15,16 | Occipital lobe_medial |
| 49,50 | SOG | Superior occipital gyrus | 17,18 | Occipital lobe_lateral |
| 51,52 | MOG | Middle occipital gyrus | 17,18 | Occipital lobe_lateral |
| 53,54 | IOG | Inferior occipital gyrus | 17,18 | Occipital lobe_lateral |
| 55,56 | FFG | Fusiform gyrus | 15,16 | Occipital lobe_medial |
| 57,58 | PoCG | Postcentral gyrus | 1,2 | Central region |
| 59,60 | SPG | Superior parietal gyrus | 17,18 | Parietal lobe_lateral |
| 61,62 | IPL | Inferior parietal, but supramarginal and angular gyri | 17,18 | Parietal lobe_lateral |
| 63,64 | SMG | Supramarginal gyrus | 17,18 | Parietal lobe_lateral |
| 65,66 | ANG | Angular gyrus | 17,18 | Parietal lobe_lateral |
| 67,68 | PCUN | Precuneus | 19,20 | Parietal lobe_medial |
| 69,70 | PCL | Paracentral lobule | 19,20 | Frontal lobe_medial |
| 71,72 | CAU | Caudate nucleus |  | Basal ganglia |
| 73,74 | PUT | Lenticular nucleus, putamen |  | Basal ganglia |
| 75,76 | PAL | Lenticular nucleus, pallidum |  | Basal ganglia |
| 77,78 | THA | Thalamus |  | Basal ganglia |
| 79,80 | HES | Heschl gyrus | 21,22 | Temporal lobe |
| 81,82 | STG | Superior temporal gyrus | 21,22 | Temporal lobe |
| 83,84 | TPOsup | Temporal pole: superior temporal gyrus | 13,14 | Limbic lobe |
| 85,86 | MTG | Middle temporal gyrus | 21,22 | Temporal lobe |
| 87,88 | TPOmid | Temporal pole: middle temporal gyrus | 13,14 | Limbic lobe |
| 89,90 | ITG | Inferior temporal gyrus | 21,22 | Temporal lobe |

**Table S2** Regions of interest for the cognitive-specific functional BG-cortical network.

| Index | Region | BA | Abbr. | Side | MNI coordinate | | |
| --- | --- | --- | --- | --- | --- | --- | --- |
|  |  |  |  |  | x | y | z |
| 1 | Caudate head |  | Cau.head.L | L | -12 | 8 | 5 |
| 2 | Caudate head |  | Cau.head.R | R | 10 | 8 | 5 |
| 3 | Red nucleus |  | RN.R | R | 6 | -20 | -8 |
| 4 | Thalamus (medial dorsal nucleus) |  | Tha.L | L | -8 | -19 | 10 |
| 5 | Thalamus (medial dorsal nucleus) |  | Tha.R | R | 10 | -31 | -2 |
| 6 | Insula | 13 | INS.L | L | -32 | 20 | 8 |
| 7 | Insula | 13 | INS.R | R | 32 | 20 | 5 |
| 8 | Cingulate gyrus | 32 | CG.L | L | -2 | 18 | 46 |
| 9 | Middle frontal gyrus | 6 | MFG.BA6.L | L | -30 | -7 | 61 |
| 10 | Precentral gyrus | 6 | PreCG.L | L | -42 | -4 | 48 |
| 11 | Middle frontal gyrus | 6 | MFG.BA6.R1 | R | 42 | 31 | 36 |
| 12 | Middle frontal gyrus | 6 | MFG.BA6.R2 | R | 44 | 7 | 35 |
| 13 | Middle frontal gyrus | 6 | MFG.BA6.R3 | R | 34 | 29 | 34 |
| 14 | Middle frontal gyrus | 9 | MFG.BA9.R1 | R | 32 | 55 | 9 |
| 15 | Middle frontal gyrus | 9 | MFG.BA9.R2 | R | 36 | 46 | 22 |
| 16 | Middle frontal gyrus | 46 | MFG.BA46.R | R | 42 | 30 | 15 |
| 17 | Inferior frontal gyrus | 47 | IFG.L | L | -46 | 20 | 3 |
| 18 | Superior parietal lobule | 7 | SPL.L | L | -30 | -62 | 42 |
| 19 | Inferior parietal lobule | 40 | IPL.L | L | -57 | -45 | 26 |
| 20 | Inferior parietal lobule | 40 | IPL.R | R | 34 | -51 | 39 |
